# Extramammary findings on breast MRI: prevalence and imaging characteristics favoring malignancy detection: a retrospective analysis

**DOI:** 10.1186/s12957-016-0865-x

**Published:** 2016-04-21

**Authors:** Soung Moon Yang, Sung Hun Kim, Bong Joo Kang, Byung Joo Song

**Affiliations:** Department of Radiology, College of Medicine, Seoul St. Mary’s Hospital, The Catholic University of Korea, Banpo-daero 222, Seocho-gu, Seoul, 137-701 Republic of Korea; Department of General Surgery, College of Medicine, Seoul St. Mary’s Hospital, The Catholic University of Korea, Seoul, Republic of Korea

## Abstract

**Background:**

Little study of the extramammary finding of breast MRIs has been done with only descriptive work of the prevalence of location and malignancy. The purpose of the present study was to assess the prevalence, the location, and the imaging characteristics of the incidentally detected extramammary findings on breast MRI and to determine potential malignant characteristics.

**Methods:**

The study evaluated extramammary findings in 109 patients who underwent breast MRI for the staging of breast cancer and for the follow-up of post-therapy. Prevalence, the location, clinicopathologic findings of breast cancer size, metastasis, and MRI characteristics were evaluated retrospectively. Malignancy of extramammary findings was determined based on the pathologic examinations and diagnostic images.

**Results:**

One hundred forty-nine incidental findings were detected in 109 (4.6 %) of 2361 patients, and 69 cases were confirmed or considered to be malignant. The most common site was the bone (43/149, 28.9 %) with malignancy found in 30 (69.8 %) of 43 bone lesions. Less frequent tumor locations were the liver (22.1 %), lung (21.5 %), pleura or chest wall (10.1 %), mediastinum (6.7 %), supraclavicular lymph nodes (LNs) (6.0 %), and others (4.7 %). Findings of significant relevance with malignancy of the extramammary findings included bigger size of breast cancer, presence of LN metastasis, and distant metastasis (*P* < 0.01). Lesions showing iso- or hypo signal intensity (SI) on T2-weighted imaging (T2WI) (*P* = 0.000), contrast enhancement (*P* = 0.000), high SI on diffusion-weighted imaging (DWI) (*P* = 0.049), low SI on apparent-diffusion-coefficient map relative to DWI (*P* = 0.000), and multiplicity (*P* = 0.000) of the extramammary finding were significantly related to malignancy.

**Conclusions:**

Extramammary findings on breast MRI are not rare. Clinicopathologic features of the breast cancer and MRI features of extramammary findings could be useful in estimating the malignancy of the incidental extramammary finding.

## Background

MRI is well known for its high sensitivity in the detection and evaluation of the extent of breast cancer [1–4]. With the use of 3.0-T MRI, which improves signal-to-noise ratio and provides better resolution than does 1.5-T MRI at the same time, more specificity for evaluating the morphology of the lesions was attained [5–7]. In the context of high soft tissue contrast and spatial resolution, whole-body MRI has higher sensitivity for detecting liver, bone, or brain metastases in metastatic cancers such as breast cancer [8, 9]. In breast cancer, whole-body MRI has shown similar sensitivity for the detection of loco-regional recurrence and distant metastasis as positron emission tomography-computed tomography (PET-CT) [10].

When reading a breast MRI, evaluation of the breast tissue, chest wall, skin, and axillary lymph nodes (LNs) should be performed primarily. However, all other anatomical structures in the field of view (FOV) such as the neck, lung, mediastinum, spine, rib, sternum, and upper abdomen should not be overlooked because these will be the potential site of metastasis. Dietzel et al. has shown sufficient diagnostic accuracy detecting distant metastasis by simply extending the FOV to the hyoid bone and upper pelvis with a coronal sequence T2 half-Fourier acquisition single-shot turbo spin-echo image [11].

Until now, there has been little study of the extramammary finding of breast MRIs. Only descriptive work of the prevalence of location and malignancy has been made [1, 12–14].

The purpose of this article is to retrospectively assess the frequency, location, and MRI imaging findings of incidentally detected extramammary findings on breast MRI and to evaluate MRI imaging characteristics that may suggest malignant lesions.

## Methods

### Subjects

We retrospectively reviewed radiology reports of breast MRI studies performed from 2009 to December 2013. We reviewed a total 3296 images from 2361 patients who had breast MRI for the staging of known breast cancer or follow-up studies after systemic chemotherapy, neoadjuvant chemotherapy, or post-operation. MRI images reporting any extramammary findings, other than breast lesion, intramammary LNs, internal mammary LNs, and axillary LNs, were included. If the same extramammary finding was found on follow-up MRI, only the initial MRI finding was analyzed and counted. Of the 2361 patients, a total of 155 findings of 114 patients were found in the radiologic reports. Six findings were excluded: two findings from two patients who had breast MRI for screening and had no breast tumor; two findings of a patient, who had breast MRI for malignancy of unknown origin that was confirmed to be lymphoma; and two findings of two patients, who had insufficient diagnostic study to determine the malignancy of the extramammary lesion.

A total of 109 patients with 149 extramammary findings met the inclusion criteria and were included in the analysis. Of these 109 patients, 20 patients had breast MRI for follow-up after operation (Breast Imaging Reporting and Data System, or BI-RADS, category 2 or 3); 69 patients had MRI for known malignancy before surgery (BI-RADS category 6); 13 patients had follow-up breast MRI after systemic therapy (*n* = 7) and neoadjuvant chemotherapy (*n* = 6); and 7 patients had breast MRI for the evaluation of the confirmed post-operative recurrence (BI-RADS category 6).

### MRI protocol

MR imaging was performed in a prone position using a dedicated bilateral breast surface coil. Imaging with a 3-T MRI system (Verio; Siemens Healthcare, Erlangen, Germany) was obtained using the following sequences: (1) an axial, turbo spin-echo T2-weighted-imaging (T2WI) sequence with a TR/TE of 4530/93, a flip angle of 80°, 34 slices, an FOV of 320 mm, a matrix size of 576 × 403, 1 number of excitations (NEX), a slice thickness of 4 mm, and an acquisition time of 2 min 28 s; (2) axial diffusion-weighted imaging (DWI) with two sequences (i.e., single-shot echoplanar image (ss-EPI) or readout segmented EPI (rs-EPI)) (*b* values 0 and 750 s/mm^2^, TR/TE 9800/87 ms and 5600/55 ms, respectively; FOV 340 × 117 mm and 360 × 180 mm, respectively; matrix size 192 × 82; slice thickness 4 mm; acquisition time 2 min 47 s and 2 min 31 s, respectively; 5 readout segments for rs-EPI). Apparent diffusion coefficient (ADC) maps were calculated automatically by using MRI software from the DWI images; (3) pre- and post-contrast, axial T1-weighted flash three-dimensional volumetric interpolated brain examination (VIBE) sequences with a TR/TE of 4.4/1.7, a flip angle of 10°, a slice thickness of 1.2 mm, and an acquisition time of 1 min. The images were obtained before and at 10, 70, 130, 190, 250, and 310 s after an injection of contrast agent gadolinium DTPA (Gd-DTPA, 0.1 mmol/kg Gadovist; Bayer Schering Pharma, Berlin, Germany). Imaging performed with a 1.5-T MRI system (Signa; GE Medical Systems; Milwaukee, WI, USA) was conducted using the following sequences: (1) axial, fat-suppressed, fast spin-echo T2WI (TR/TE = 4000/85, a flip angle of 90°, 30 slices, an FOV of 240 mm, a matrix of 256 × 224, a NEX of 2, a 3-mm slice thickness with a 0.1 mm slice gap, and an acquisition time of 2 min 56 s); (2) axial DWI with single-shot echo planar imaging (EPI) (*b* = 0 and 1000 s/mm^2^, TR/TE = 6000/75, a FOV of 360 mm, a matrix of 128 × 128, 2 NEX, a 4-mm slice thickness with a 1-mm slice gap, and an acquisition time of 1 min 30 s); and (3) pre- and post-contrast, axial spin-echo T1-weighted imaging (T1WI) (TR/TE = 6.2/3.1, a flip angle of 10°, 2.6-mm section thickness, an FOV of 300 mm, a matrix of 256 × 192, and an acquisition time of 1 min 31 s) obtained before and 91, 192, 273, 364, and 455 s after the rapid bolus injection of Gd-DTPA.

Post-processing manipulation included the production of subtraction, multiplanar reconstruction of sagittal image, and maximum-intensity-projection (MIP) images.

### Image analysis

Images were evaluated by a radiologist with 9 years of experience in the field of evaluation of breast MRI.

Location was categorized into seven organs (liver, lung, bone, mediastinum, pleural or chest wall, supraclavicular LN, or other uncategorized location). Multiplicity was defined as having more than one separate lesion with a same imaging feature in the same organ. Signal intensity (SI) on T1WI and T2WI, DWI, and ADC map was categorized into high, iso-, or low intensity compared with surrounding normal tissue intensity.

Size of the lesion was measured as the longest diameter in axial section on T2WI or contrast-enhanced T1WI.

### Determination of malignancy

We concluded presence of a malignant lesion if it were confirmed by pathologic examination. If pathologic examination was not made, we considered malignancy based on other diagnostic examinations and follow-up imaging studies. CT, PET-CT, ultrasound, and skeletal scintigraphy were used, and malignancy was suggested if increased uptake on PET-CT correlated in location corresponding with abnormal findings on MRI or the lesions became larger during the follow-up periods or decreased in size after chemotherapy or radiation treatment [15].

### Statistical analysis

Malignancy of the extramammary findings was correlated with the clinicopathologic findings and MRI imaging characteristics using univariate logistic regression analysis. Pearson’s chi-square test was used to assess the relationship between malignancy and each MRI imaging characteristic or multiplicity. The data were analyzed using the SPSS software version 21.0 (Statistical Package for Social Sciences, SPSS, Chicago, IL, USA). Statistical significance was defined as *P* < 0.05.

## Results

**Fig. 1:**
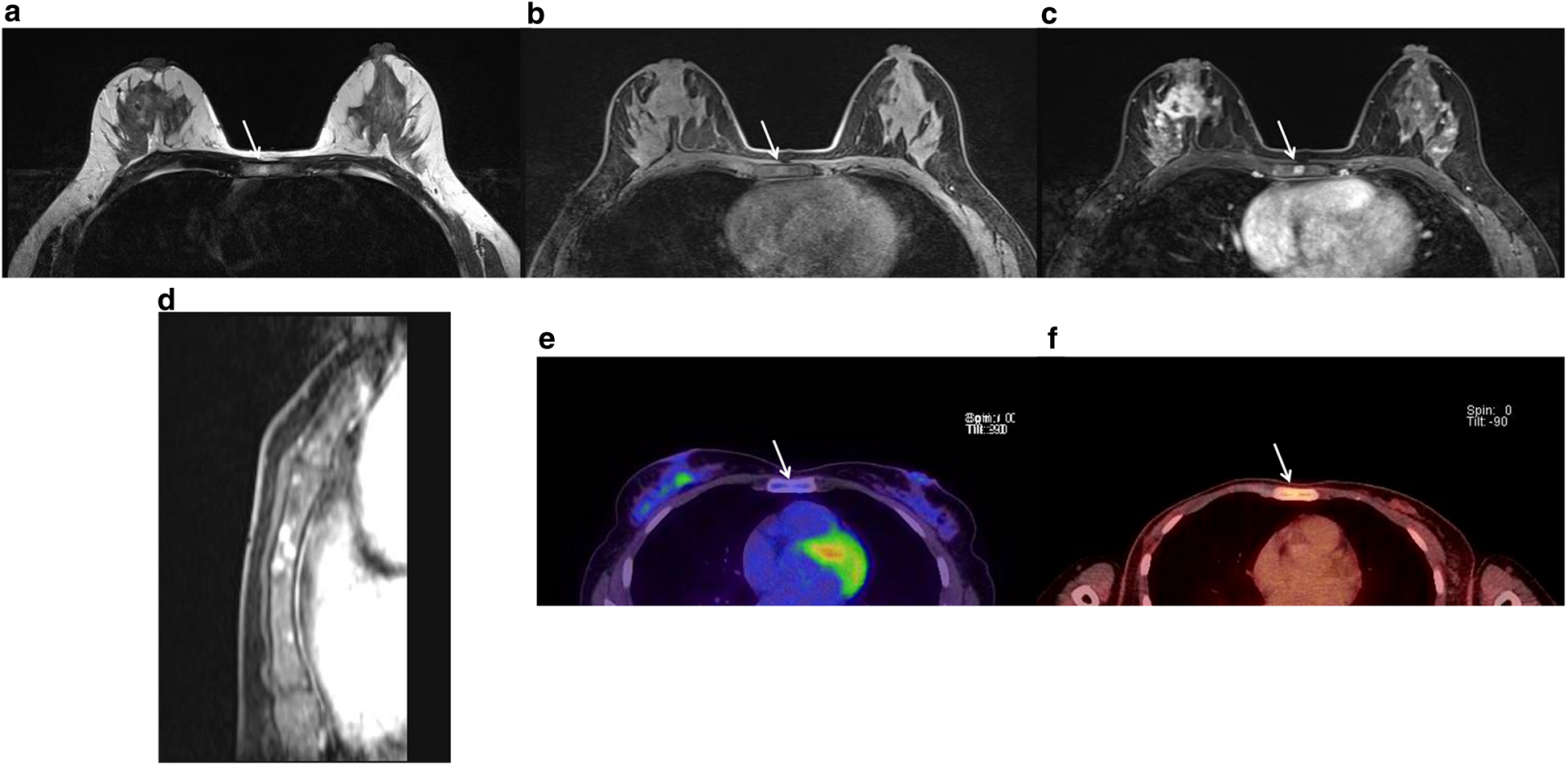
Early detection of bone metastases with MRI. Case of a 43-year-old female patient diagnosed with stage I breast cancer. Multiple sternum lesions (*arrow*) shown as hyperintense nodules on T1WI (**a**), T2WI (**b**), with positive contrast enhancement (**c**), and multiplicity (**d**) on contrast-enhanced T1WI. On initial PET/CT (**e**), no definite uptake was noted, but 7 months later, FDG uptake was seen on follow-up PET/CT (**f**)

**Fig. 2:**
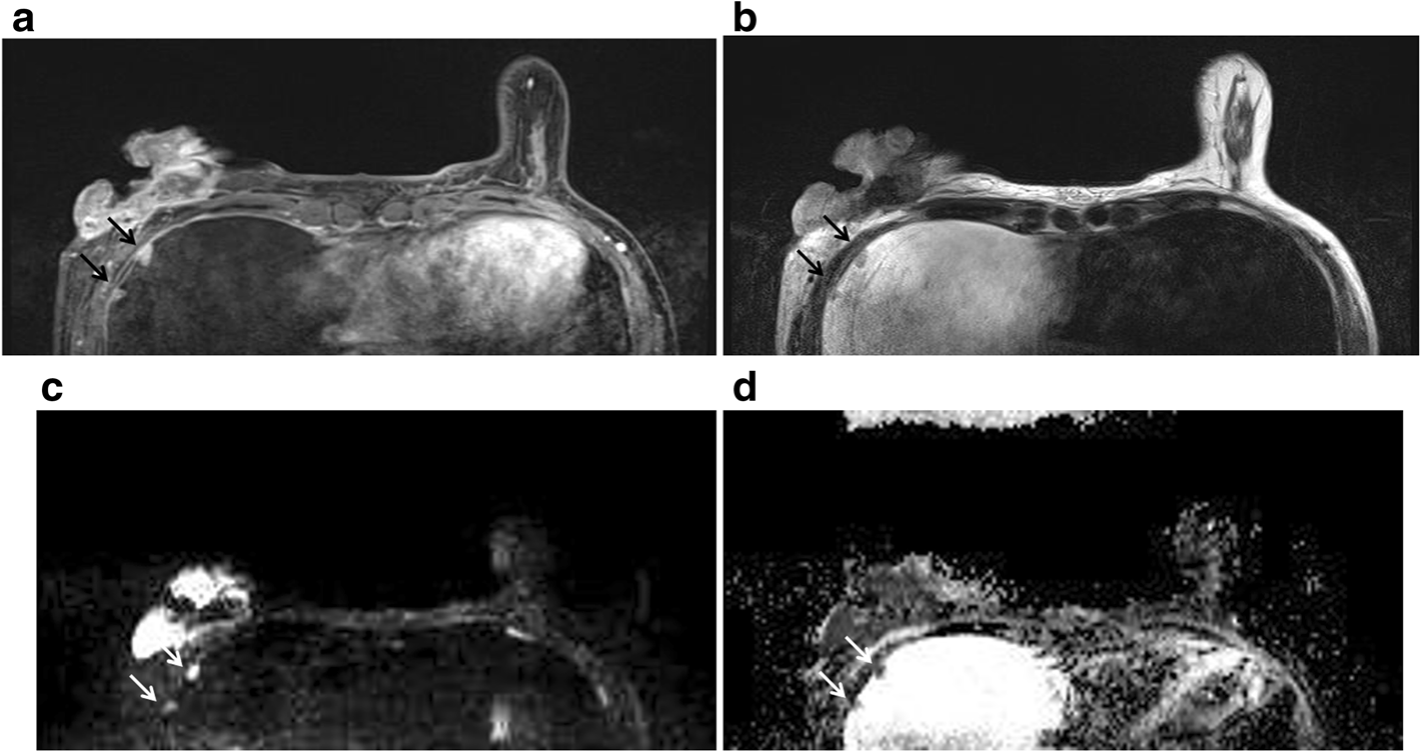
Multiple pleural metastases. Pre-operative breast MRI of a 46-year-old female patient with confirmed right breast cancer on biopsy. Multiple pleural nodules (*arrows*) showing contrast enhancement (**a**), isointense on T2WI (**b**), and definite diffusion restriction (**c**, **d**). These lesions were confirmed as pleural metastases in follow-up PET/CT and chest CT

A total of 109 patients was found to have 149 incidental extramammary findings (Table 1). Incidental finding was found in the bone (43/149, 28.9 %), liver (33/149, 22.1 %), lung (32/149, 21.5 %), pleura or chest wall (15/149, 10.1 %), mediastinum (10/149, 6.7 %), supraclavicular LN (9/149, 6.0 %), and other sites (6/149, 4.7 %). Total number of benign lesions was 80 (53.7 %), and malignancy was 69 (46.3 %). The most common site of malignant lesion was the bone (30/69, 43.5 %) (Fig. 1). Other common sites of malignant lesions were the lung (11/69, 15.9 %), pleura or chest wall (8/69, 11.6 %), supraclavicular LN (8/69, 11.6 %), liver (7/69, 10.1 %), mediastinum (4/69, 5.8 %), and other sites (1/69, 1.4 %). The site with the highest malignancy rate was the supraclavicular LN with 88.9 % (8/9), followed by bone malignancy, with a rate of 69.8 % (30/43) (Fig. 1), pleura or chest wall 53.3 % (8/15) (Fig. 2), lung 34.3 % (11/32), liver 21.2 % (7/33), mediastinum 40 % (5/10), and others (1/7, 14.3 %).

**Table 1:** Incidence of extramammary findings according to location

Location	Benign	Malignancy	Malignancy rate	Incidence
Bone	13	30	30/43 (69.8)	43 (28.9)
Liver	26	7	7/33 (21.2)	33 (22.1)
Lung	21	11	11/32 (34.3)	32 (21.5)
Pleura and chest wall	7	8	8/15 (53.3)	15 (10.1)
Mediastinum	6	4	4/10 (40.0)	10 (6.7)
Supraclavicular lymph node	1	8	8/9 (88.8)	9 (6.0)
Other	6	1	1/7 (14.3)	6 (4.7)
Total	80	69	69/149 (46.3)	149

Numbers in parentheses are percentage

The relevance of patient age and clinicopathologic findings of breast cancer and the malignancy of the extramammary findings is summarized in Table 2. Patient’s age had no significant relevance (*P* = 0.569, odds ratio, OR = 0.99). Larger tumor size (*P* = 0.006, OR = 1.37) (Fig. 2), existence of LN metastasis (*P* = 0.000, OR = 3.99), existence of distant metastasis (*P* = 0.000, OR = 32.33), and higher TNM stage (I/II vs. III/IV, *P* = 0.000, OR = 19.18) had significant relation with malignancy of the extramammary finding.

**Table 2:** Univariate logistic regression analysis of clinicopathologic characteristics of breast cancer for differentiating malignancy from the benign extramammary finding

Variables	Benign (percentage)	Malignancy (percentage)	Univariate logistic regression	
			Odds ratio (95 % CI)	*P* value
Age	56.29 ± 12.68	54.97 ± 15.45	0.99 (0.97, 1.01)	0.569
Tumor size (cm)	2.69 ± 1.89	4.23 ± 2.58	1.37 (1.09, 1.71)	0.006
Lymph node metastasis	23/66 (34.8)	16/26 (61.5)	3.99 (1.99, 8.00)	0.000
Distant metastasis	7/71 (9.9)	48/62 (77.4)	32.33 (12.13, 86.14)	0.000
TNM stage (I/II vs. III/VI)			19.18 (7.74, 47.54)	0.000
Stage I	23/71 (32.4)	2/62 (3.2)		
Stage II	30/71 (42.2)	6/62 (9.7)		
Stage III	13/71 (18.3)	7/62 (11.3)		
Stage IV	5/71 (7.0)	47/62 (75.8)		

*CI* confidence intervals

**Fig. 3:**
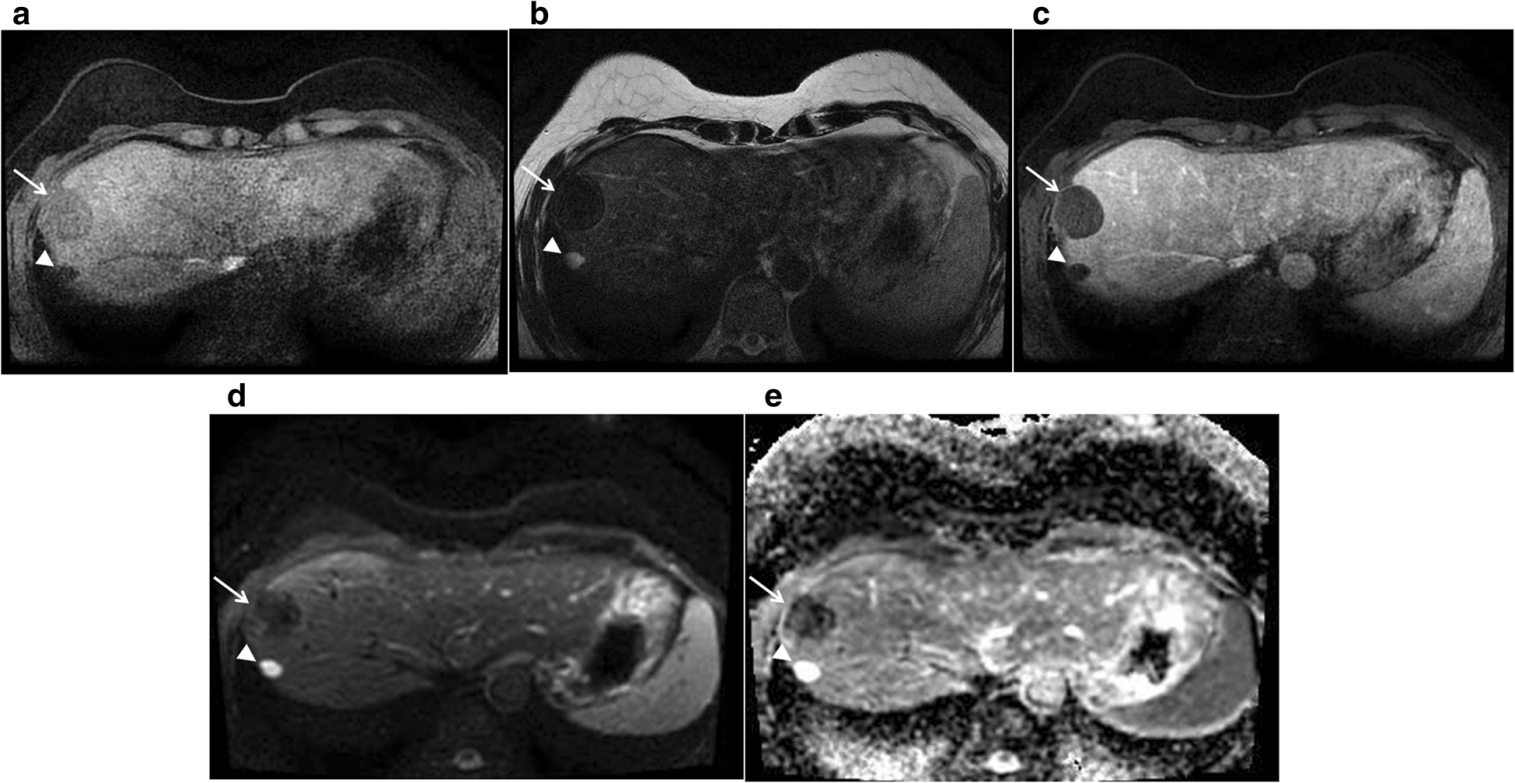
Benign liver mass. Case of a 49-year-old female patient with two lesions. A solid mass (*arrow*) with a small cyst (*arrow head*) on the posterior aspect in is seen in the right hepatic lobe. The lesion shows hypointense on T1WI (**a**) and T2WI (**b**), with no definite contrast enhancement (**c**), and no diffusion restriction (**d**, **e**). This was thought to be a dense calcific benign mass, and additional follow-up MRIs and CTs for 2 years showed no interval change, so we were able to confirm it as a benign lesion

MRI imaging characteristics of the extramammary findings between benign and malignancy are shown in Table 3. Multiplicity of the finding (*P* = 0.000, OR = 5.13), iso-/low SI on T2WI (*P* = 0.028, OR = 0.46), positive contrast enhancement (*P* = 0.000, OR = 7.39), high SI on DWI (*P* = 0.049, OR = 8.18), and low SI on ADC map (*P* = 0.000, OR = 33.70) were significantly related to malignancy of the finding (Figs. 1, 2, and 3). Size of the finding (*P* = 0.159, OR = 0.88) and high SI on T1WI (*P* = 0.574, OR = 0.82) have shown no significant relation with the malignancy of the finding.

**Table 3:** Univariate analysis for MRI findings of extramammary finding

Variables	Case numbers	Benign (percentage)	Malignancy (percentage)	Univariate logistic regression	
				Odds ratio (95 % CI)	*P* value
Multiplicity	149			5.13 (2.54, 10.37)	0.000
Single	76	55/76 (72.4)	21/76 (27.6)		
Multiple	71	24/71 (33.8)	47/71 (66.2)		
Size (cm)	149	2.47 ± 1.98	1.99 ± 2.07	0.88 (0.74, 1.04)	0.159
T1 signal intensity	141			0.82 (0.41, 1.64)	0.574
Hyperintense	52	30/52 (57.7)	22/52 (42.3)		
Iso/hypointense	89	47/89 (51.6)	42/89 (46.1)		
T2 signal intensity	138			0.46 (0.23, 0.92)	0.028
Hyperintense	86	53/86 (61.6)	33/86 (38.4)		
Iso/hypointense	52	22/53 (42.3)	30/52 (57.7)		
Contrast enhancement	146			7.39 (2.86, 19.08)	0.000
Presence	106	46/106 (43.4)	60/106 (56.6)		
Absence	40	34/40 (85.0)	6/40 (15.0)		
DWI signal intensity	91			8.18 (1.00, 67.03)	0.049
Hyperintense	80	44/80 (55.0)	36/80 (45.0)		
Iso/hypointense	11	10/11 (90.9)	1/11 (9.1)		
Apparent diffusion coefficient map	81			38.70 (10.76, 139.20)	0.000
Hyperintense	32	5/32 (15.6)	27/32 (84.4)		
Iso/hypointense	49	43/49 (87.8)	6/49 (12.2)		

*CI* confidence intervals, *DWI* diffusion-weighted imaging

Most malignant extramammary findings showed high intensity on DWI and low SI on ADC map (Table 4).

**Table 4:** Characteristics of malignant extramammary findings on diffuse-weighted images

Location	DWI signal intensity		ADC map	
	Hyperintense	Iso/hypointense	Low SI	High SI
Bone	17	1	13	3
Liver	3	0	3	0
Lung	8	0	6	1
Pleura and chest wall	5	0	3	2
Mediastinum	2	0	1	0
Supraclavicular lymph node	0	0	0	0
Other	1	0	1	0

## Discussion

Recently, multi-channel whole-body MR systems using 3-T MR became available and are known to have high sensitivity for distant metastasis and detecting LN involvement [8–10]. Accordingly, MRI can be useful for detecting extramammary malignant lesions included in the FOV of breast MRI, in addition to other benign lesions. Our results reinforce the importance of extramammary findings of breast MRI that had been noted in an earlier study [13].

Overall, incidental extramammary findings were observed in 155 (4.7 %) of 3296 dynamic contrast-enhanced breast MRI exams. We have not counted the same extramammary findings observed repeatedly on follow-up studies, so the exact prevalence would be 4.8 % (*n* = 114) of 2361 patients. Nevertheless, the prevalence of our study was lower compared with other prior studies, which reported prevalence as 9 % [12] and 17 % [1]. Our study showed relatively low prevalence (49.5 %) of malignancy (50/101) in patients examined for pre-operative staging compared with a previous study that showed 81 % prevalence [12]. One factor might be that we have excluded the number of the same findings on follow-up studies. Furthermore, we only retrospectively reviewed the radiology reports, which may have ignored trivial and obvious benign lesions.

Common sites of malignancy were the supraclavicular LN, bone, lung, pleura or chest wall, and mediastinum, ordered by frequency. This corresponds well with a prior study of common breast cancer metastasis sites, which are known to be the bone, lung, liver, pleura, peritoneum, and others [16] or the bone, lung/pleural, chest wall/skin, nodes, liver, and others [17]. Our study also has results similar to other studies of incidental extramammary findings [1, 12, 13]. The liver was the most frequent site of benign lesions in our study. Former studies also showed highest prevalence of incidental extramammary findings in the liver, with low malignancy rate [1, 12–14].

Our study showed that the (1. higher TNM stage), (2. bigger tumor size), (3. existence of LN metastasis), (4. existence of distant metastasis) are all significantly related to malignancy of extrammamary finding. This is consistent with our understanding of cancer mechanism.

Our study also showed that multiplicity, positive contrast enhancement, iso- or low SI on T2WI, high SI on DWI, and low SI on ADC map are significantly related to malignancy of extramammary findings. Multiplicity is a common feature of hematogenous metastasis [18, 19], and multiplicity raises the suspicion for malignancy in lung nodules [18]. Although the probability of a hepatic lesion being malignant is less than 20 % [14, 20], further studies are required when suspicious characteristics are observed on the MRI, such as the presence of multiplicity, indistinct margins, or rim enhancement [21]. Our study showed that the positive enhancement of the liver nodule was related to malignant finding (*P* = 0.003). However, multiplicity of liver nodules showed no significant relationship with malignancy (*P* = 0.174).

Some previous articles mentioned that bone metastasis of breast cancer is commonly observed as high SI on T2WI [14]. Another study showed that mixed characteristics on T2WI [22] are significantly related to bone metastasis of breast cancer, and our study also showed similar results: high SI 37 %, iso- to low SI 63 %.

DWI is well known for its high sensitivity and specificity of detecting metastasis and malignancies [23–26]. Our study also showed that metastatic lesions mostly show high SI on DWI with low SI on ADC map (Table 4). Our study showed significance of SI on ADC map in comparison with that on DWI by means of determining malignancy of incidental findings, a comparison that was never studied before, to our knowledge. Our findings demonstrated effectiveness of DWI in determining malignancy of incidental finding. One of our cases showed that DWI can detect small metastatic lesions before PET-CT, as shown in Fig. 3. However, it is also important to check other features because DWI alone shows low specificity for the detection of regional or metastatic lesions [24].

There are several limitations to this study. First, it was a retrospective study. Second, we only searched images through radiologic reports; thus, the possibility exists that many incidental findings were not counted in our study because they were not mentioned in the radiologic report due to low significance or benignity. Third, only two cases were confirmed by pathologic evaluation, and 67 other cases were considered metastatic on the basis of other follow-up confirmation examinations. We believe additional studies with prospective design and pathologic confirmation will give more accurate information for determining malignancy.

## Conclusions

In conclusion, incidental extramammary findings on breast MRI should not be neglected. A significant portion of the findings can be malignant, and that could change the diagnostic work-up or treatment plan. In addition to imaging findings such as multiplicity, SI on T2WI and contrast enhancement on T1WI, DWI, and ADC map can be reliable and effective tool to determine malignancy.

## Ethics approval and consent to participate

This retrospective study was approved by the institutional review board (Catholic Medical Center, Seoul, Korea), and the requirement for the informed consent was waived.

## Abbreviations

ADC: apparent diffusion coefficient
DWI: diffusion-weighted imaging
FOV: field of view
LN(s): lymph node(s)
PET-CT: positron emission tomography-computed tomography
SI: signal intensity
T1WI: T1-weighted imaging
T2WI: T2-weighted imaging
